# Systematic Review of Clinical Applications of CAD/CAM Technology for Craniofacial Implants Placement and Manufacturing of Nasal Prostheses

**DOI:** 10.3390/ijerph18073756

**Published:** 2021-04-03

**Authors:** Waqas Tanveer, Angela Ridwan-Pramana, Pedro Molinero-Mourelle, Jan Harm Koolstra, Tymour Forouzanfar

**Affiliations:** 1 Department of Oral and Maxillofacial Surgery, Amsterdam UMC, 1081 HZ Amsterdam, The Netherlands; 2 Center for Special Care in Dentistry, Department of Maxillofacial Prosthodontics, Stichting Bijzondere Tandheelkunde, 1081 LA Amsterdam, The Netherlands; a.ridwan@amsterdamumc.nl; 3 Department of Oral and Maxillofacial Surgery/Oral Pathology and 3D Innovation Lab, Amsterdam UMC, 1081 HZ Amsterdam, The Netherlands; t.forouzanfar@amsterdamumc.nl; 4 Department of Reconstructive Dentistry and Gerodontology, School of Dental Medicine, University of Bern, CHE 3012 Bern, Switzerland; pedro.molineromourelle@zmk.unibe.ch; 5 Department of Oral Cell Biology and Functional Anatomy, Academic Centre for Dentistry Amsterdam (ACTA), University of Amsterdam and VU University, 1081 LA Amsterdam, The Netherlands; j.koolstra@amsterdamumc.nl

**Keywords:** nasal prosthesis, digital planning, digital workflow, craniofacial implants, guided implants surgery

## Abstract

The aim of this systematic review was to gather the clinical and laboratory applications of CAD/CAM technology for preoperative planning, designing of an attachment system, and manufacturing of nasal prostheses. According to Preferred Reporting Items for Systematic Reviews and Meta-Analyses (PRISMA) guidelines, an electronic search was carried out. Only human clinical studies involving digital planning for the rehabilitation of facial defects were included. A total of 21 studies were included with 23 patients, which were virtually planned through different planning software. The most common preoperative data for digital planning were CT scans in nine cases, CBCT in six cases, and laser scans in six cases. The reported planning softwares were Mimics in six cases, Geomagic Studio software in six cases, ZBrush in four cases, and Freeform plus software in four cases. Ten surgical templates were designed and printed to place 36 implants after digital planning, while post-operative assessment was done in two cases to check the accuracy of planned implants. Digital 3D planning software was reported for presurgical planning and craniofacial implants placement, fabrication of molds, designing of implants, designing of retentive attachments, and printing of silicone prostheses. Digital technology has been claimed to reduce the clinical and laboratory time; however, the equipment cost is still one of the limitations.

## 1. Introduction

Maxillofacial defects can be caused by genetic malformations, ablative tumor surgery, and trauma. These defects require immediate intervention to improve the quality of life of a patient [1,2,3]. Nasal defects most significantly affect the esthetics and psychology of patients due to their central location on the face. Treatment options to rehabilitate these patients include surgical reconstruction or prosthetic rehabilitation [4,5,6,7,8,9]. Surgical reconstruction can be done with a bilobed flap, nasolabial flap, forehead flap, septal mucosal flap, S-shaped rotation flap, croissant-shaped flaps, modified V-Y flaps, radial forearm free flaps, and titanium mesh [10]. However, surgical reconstruction involving the entire nasal cavity poses significant challenge to reconstructive surgeons; therefore, it is currently only performed with satisfactory results in a few specialized medical centers around the world [11]. In contrast, the prosthetic rehabilitation of such defects is more commonly performed by covering the defect with silicone prosthesis while maintaining the patency of airway. Nasal prostheses, similar to various other facial prostheses, can be retained by medical adhesives, mechanical attachments, anatomical undercuts, and craniofacial implants [12]. Among different retentive options, craniofacial implants have been documented to provide the optimum retention and stability of prostheses [13]. The most common implants locations to retain a nasal prosthesis are the anterior maxilla (floor of nasal cavity), zygomatic bone, and glabella [14,15]. The success rate of implant in the glabella region is lower as compared to anterior maxilla, which is probably due to the poor blood supply and density of bone in this region [15].

The planning and placement of craniofacial implants for nasal defects can be challenging due to the presence of natural teeth in the anterior maxilla and limited quantity of bone around paranasal sinuses [15]. Therefore, planning and precision in implants placement is critically important to avoid sensitive anatomical structures. Conventionally, craniofacial implants are guided by wax prototypes, which are duplicated into hard acrylic templates [16]. Ultimately, computed tomography (CT) template measurements can help to locate the precise location for implants placement [17]. These conventional surgical templates help to precisely mark the planned implant location over the skin before initial incision is made; however, once the flap is elevated, the chances of error increase. Thus, conventional soft tissue-supported guides neither provide surgeons with any direct reference to the quantity or quality of underlying bone nor the appropriate angulation or depth of implants placement.

Integration of computer-aided design (CAD) and computer-aided manufacturing (CAM), have brought revolution in the field of maxillofacial prosthetics during the last decade [18], with benefits including precise planning, predictable outcome, considerably less clinical and laboratory time, and yet an economical cost of prostheses [9,19]. Digital planning software have been used for surgical assistance intra-operatively [20]. These software gather the data from radiographic investigations, surface scans, and study models to provide the exact information about the height and width of underlying bone along with associated anatomical structures. These digitally designed surgical templates improve the accuracy of implants placement in terms of precise location, depth, and inclination of implants. Accurate measurements from the preoperatively planned position to post-operative implant location are measured with the help of CT scan, cone beam computed tomography (CBCT) scan, and superimposition method in software by using best fit alignment function [9,19]. The extent of precision and accuracy varies among different planning software. However, these digital guides do have the disadvantage of requiring a larger area of exposure to allow stable placement over the bone surface [20]. The aim of this study is to gather the clinical data to respond to the following question: In patients with nasal defects, what are the technical and clinical applications of CAD/CAM technology for the preoperative planning, designing, and manufacturing of nasal prostheses?

## 2. Experimental Section

A systematic review was conducted in accordance with a protocol based in all Preferred Reporting Items for Systematic Reviews and Meta-Analyses (PRISMA) [21] in order to assess the PICO (patients, investigation, comparison, outcome) question: In patients with nasal defects, what are the technical and clinical applications of CAD/CAM technology for the preoperative planning, designing, and manufacturing of nasal prostheses?

### 2.1. Search Strategy

The electronic search was performed by entering the combination of following terms: {Prostheses AND Planning AND Guide}.

Prosthesis: (Nasal prostheses OR nose prostheses OR midface prostheses OR silicone nasal prosthesis) AND Planning: (CAD/CAM OR scanning OR digital OR software planning OR navigation OR 3D) AND Guide: (implants OR craniofacial implants OR extraoral implants OR surgical guide OR surgical template OR guided surgery OR printed guide)

### 2.2. Eligibility Criteria

**Figure 1:**
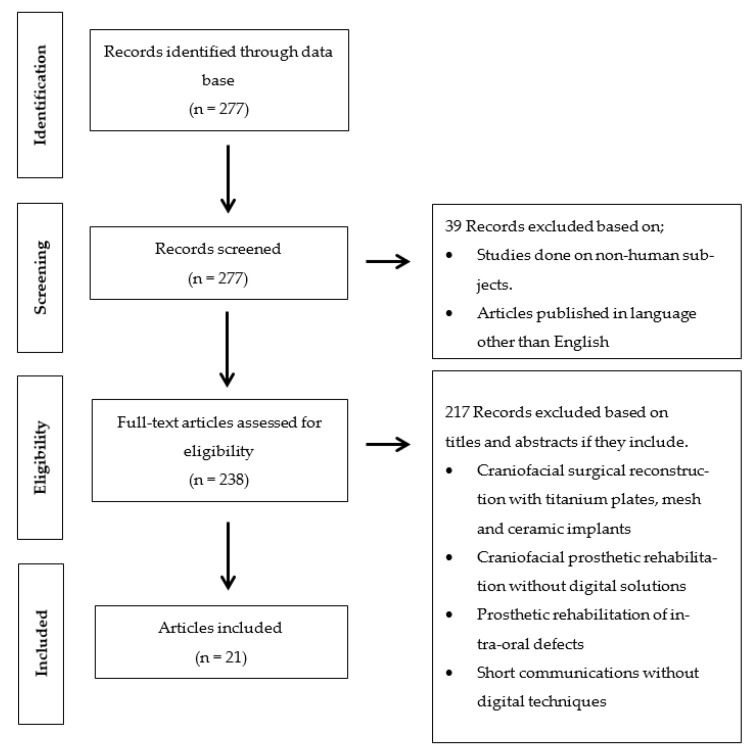
Flow-chart of studies selection process and screening methodology.

The clinical human studies, which, were published in English language from 2009 to 2020, were included in this review. Inclusion criteria involved clinical human studies, randomized control trials, cohort studies, case control studies, case series, case reports involving the digital planning software for craniofacial implants placement or fabrication of nasal prosthesis. Exclusion criteria were systematic reviews, finite element analysis (FEA), animal studies, in vitro studies, and case reports executed without digital planning software (Figure 1).

### 2.3. Source of Information

An electronic search from January 2009 to October 2020 was made on The National Library of Medicine (MEDLINE/PubMed) database.

Moreover, a manual search of the following journals from January 2009 until October 2020 was also performed: The Journal of Oral Rehabilitation, the Journal of Prosthetic Dentistry, the Journal of Prosthodontics, the International Journal of Prosthodontics, the Journal of Prosthodontic Research, Clinical Oral Implants Research, the Journal of Oral Implantology, the International Journal of Oral and Maxillofacial Implants, International Journal of Oral and Maxillofacial Surgery, Journal of Oral and Maxillofacial Surgery, Journal of Cranio-maxillo-facial surgery, Journal of Stomatology, Oral and Maxillofacial Surgery, British Journal of Oral and Maxillofacial Surgery, Implant Dentistry, and Clinical Implant Dentistry and Related Research.

### 2.4. Study Selection

The study selection was performed independently by two independent (W.T. and P.M.M.) reviewers through titles and abstracts of all identified studies through an electronic search read individually by the authors. For the studies that appeared to fulfill the inclusion criteria or those studies that had limited data in the title and abstract to reach the final decision, the full record was gathered. Disagreements among authors were resolved after discussion.

### 2.5. Data Extraction

The data from each included study were extracted according to the designed standard form: author’s name, country, year of publication, prostheses designed and/or fabricated, number of implants placed, purpose of using digital planning and printing software, names of software, material used to print template, prostheses and molds, implant’s system, and post-operative assessment (Table 1). Contact was made with the authors for possible missing data.

### 2.6. Risk of Bias in Individual Studies

Two independent reviewers (W.T. and P.M.M.) evaluated the quality of the included studies. If there were conflict of agreement on any paper, it was further evaluated by a third reviewer (A.R.P.). For the evaluation stage, the critical tools of The Joanna Briggs Institute [22] (JBI) for case series and clinical case reports were used according to the type of included articles. The bias was evaluated through a list of eight questions for the case report and 10 questions for the case series, respectively. Questions are specified in Table 2 and Table 3 regarding the risk of bias. Finally, an overall appraisal was made to determine if the risk of bias is low (included), high (excluded), or uncertain (more information needs to be sought). We considered there to be a high risk of bias if the answers “no” were ≥50%, a low risk of bias if the answers “yes” were ≥50%, and an uncertain risk of bias if the “unclear” answers were ≥50%.

## 3. Results

### 3.1. Study Selection

The literature was searched using the above-mentioned terms through the PubMed database. The flowchart of literature search and selection process is shown in Figure 1. As most of the advancement in virtual planning and printing software for maxillofacial rehabilitation has been seen since the last decade [18]; therefore, an initial search yielded 277 studies with time filter (January 2009–October 2020). A total of 39 studies were excluded through language (English) and human studies filters. Furthermore, 238 studies were screened according to the inclusion and exclusion criteria; therefore, an additional 217 studies were excluded based on their study design and rehabilitation techniques (craniofacial surgical reconstruction with titanium plates, mesh and ceramic implants, craniofacial prosthetic rehabilitation without digital solutions, prosthetic rehabilitation of intra-oral defects, and short communications without digital techniques). A total of 21 studies [9,19,23,24,25,26,27,28,29,30,31,32,33,34,35,36,37,38,39,40,41] involving 23 cases were planned and executed with digital planning software for prosthetic rehabilitation of nasal defects (Table 1). Due to the included studies’ quality and data heterogeneity, meta-analysis could not be performed.

### 3.2. Study Characteritics

#### 3.2.1. Applications of CAD/CAM Technology for Surgical and Prosthetic Purpose

The included studies had the following purposes for utilizing digital software during preoperative planning: the fabrication of surgical templates (10 cases), molds fabrication for silicone packing (5 cases), designing of substructure (3 cases), direct printing (2 cases), implants fabrication (2 cases), rapid prototyping of nose models (2 cases), creation of nose database (1 case), and fabrication of copy milled framework for nose prosthesis (1 case).

#### 3.2.2. Preoperative Planning 

Preoperative data included CT scan (9 cases), CBCT scan (6 cases), Laser scans; NextEngine Desktop, (3 cases), Laser scan; VIVID 900 (1 case), Laser scan; 3dMDface System (1 case), Laser scan; and an ATOS scanner (1 case). Digital images: 3dMDcranial system (1 case); Lava COS intra-oral scans (1 case); Stationery 3D photogrammetry images, pritiface (1 case); stationary images, G-scan (1 case). Light scan: ATOS III (1 case), structured light scan; Artec Spider; Artec 3 (1 case), and structured light scan; Rexcan 3 (1 case). 

The digital software used during preoperative planning by various case studies were Mimics (6 cases), Geomagic studio (6 cases), ZBrush (4 cases), Freeform plus (4 cases), Rapidform (3 cases), Lava COS (3 cases), 3ds Max (3 case), Novel guide software (3 cases), SimPlant Planner (2 cases), Rhinoceros (2 cases), Novel clinician; nobel Biocare Procera (1 case), epiTecture (1 case), Coral Paintshop Pro (1 case), Materialise CMF pro plan (1 case), and Amira (1 case).

#### 3.2.3. Printing Equipment Devices

Digital printers utilized after designing and planning stages were SLA systems (4 cases), Stratasys system (3 cases), DSM Desotech (1 case), Z Printer (1 case), Nobel Biocare printer (1 case), Binder jet printer (1 case), Rapid prototyping system (1 case), 3D milling system (1 case), and ACEO drop on demand printer (1 case). The most common printing materials used in included studies were ABS (3 cases), SLA resin (3 cases), titanium alloy (2 cases), cyanoacrylate resin (1 case), polyamide resin (1 case), photopolymer (1 case), thermoplastic material (1 case), acrylic resin (1 case), and silicone free of solvent (1 case).

#### 3.2.4. Guided Implants Surgery

A total of 36 implants were placed in 23 cases after digital designing and planning stages. However, no implant failure was mentioned in any case study. Additionally, post-operative assessment was reported in only two case studies. According to Ciocca L et al. [9], a post-operative CT scan revealed that apex deviation ranged from 1.17 to 2.81 mm, while the angular deviation ranged from 1.87° to 7.78°. Furthermore, Van der Meer et al. (2012) reported after CBCT assessment that all implants were placed well within the limits needed for the fabrication of an optimal prosthesis, both from a surgical and prosthodontics perspective.

### 3.3. Risks of Bias in Individual Studies

Following the criteria provided by JBI [22], the risk of bias of included studies was assessed. As shown in Table 2, the case reports authored by Ciocca et al. 2011 [9], Ciocca et al. 2010 [23], Walivaara et al. 2011 [24], Ciocca et al. 2010 [25], Toso et al. 2015 [26], Buzayan et al. 2017 [27], Dawood et al. 2012 [28] Unkovskiy et al. 2018 [29], McHutchion et al. 2019 [30], Qiu et al. 2011 [31], Reitemeier et al. 2013 [32], Grant et al. 2015 [33], Ciocca et al. 2016 [34], Palousek et al. 2014 [35], Dawood et al. 2017 [36], Neto et al. 2014 [37], Nuseir et al. 2019 [38], Vera et al. 2014 [39], Yoshioka et al. 2016 [40], Tso et al. 2015 [41], presented a low risk of bias. Furthermore, Table 3 showed the case series authored by Van der Meer et al. 2012 [19], resulting in a low risk of bias.

**Figure 2:**
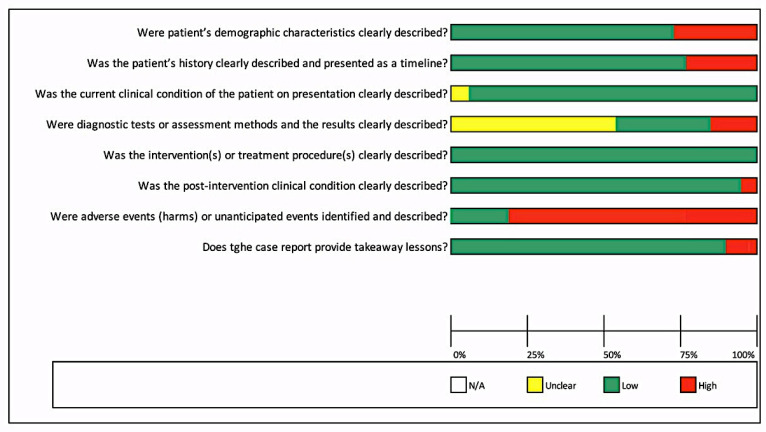
Risk of bias across included studies for case reports.

In Figure 2, it can be observed that most studies had a low risk of bias ≤ 50%, except for the question, “Were adverse events (harms) or unanticipated events identified and described?”, for which more than 75% of studies had not mentioned any adverse event or unanticipated events. While for one question, “Were diagnostic tests or assessment methods and the results clearly described?”, more than 50% of studies had not clearly mentioned the diagnostic tests or assessment methods or results of investigations.

**Figure 3:**
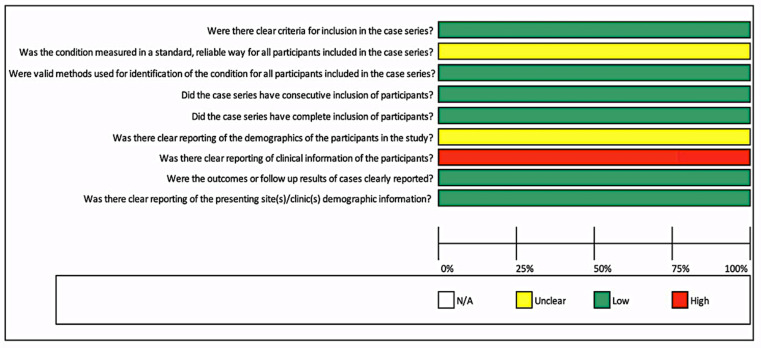
Risk of bias for case series.

Furthermore, Figure 3 showed the risk of bias for one case series. Most questions presented a low risk of bias except for one question: “Was there clear reporting of clinical information of the participants?” A high risk was observed, as no significant clinical information about patients was described. Moreover, details were unclear for two questions: “Was the condition measured in a standard, reliable way for all participants included in the case series?” and “Was there clear reporting of the demographics of the participants in the study?” Furthermore, it was not possible to perform a meta-analysis due to the quality of included studies, case series, and case reports.

## 4. Discussion

Digital planning and printing technology had opened the doors to healthcare professionals in last few decades. Since 1997, various systems for computer-guided implants placement have been available for intra-oral implants [42,43]; however, their use for craniofacial implants planning and placement has not been practiced until the last decade. In the last decade, CAD/CAM technology applications have dramatically increased due to predictable outcomes and reduced clinical and laboratory time of procedures, which enabled the patients to virtually visualize the end results preoperatively, reduced the patient’s appointments, and enabled the direct fabrication of prosthesis and surgical guides. (Table 4) Therefore, this study was aimed to gather data about the various clinical and laboratory applications of CAD/CAM technology for preoperative planning, the design of an attachment system, and the manufacturing of nasal prostheses.

The preoperative data collection is the first step during software planning; therefore, the quality and accuracy of these data significantly affect the accuracy of final outcome. Van Eijnatten et al. [44] observed the geometrical deviations up to +0.9 mm for study models, with the highest deviations noticed in CBCT-derived STL files. Errors can occur in gantry tilt, slice thickness, tube current, pitch, voltage, algorithm for image’s slices reconstruction, and patient movements as well as artifacts arising from metal prostheses [45]. The thickness of slices directly affects the volume measurements; therefore, they should be kept <1.25 mm [46,47]. In this review, the preoperative dataset consisted of CT scans, CBCT, laser scans, and photogrammetry. A total of two studies described the slice thickness of CT scans and voxel sizes of CBCT. The CBCT voxel size was found consistent at 0.3 mm, while the CT scan slice thickness was 1 mm, respectively [19,37].

The major revolution in digital planning was the integration of laser scan data and 3D radiographic images as the starting point for the design of the surgical guide. This relative integration of two entities enabled the prosthodontists and surgeons to plan the implant surgeries in chronological sequence from future prosthetic position and morphology to the proposed location of implants. In this review, 19 studies utilized 3D radiographic images and surface scans, out of which five studies made use of CT or CBCT scans along with surface scans together for preoperative planning [9,19,23,37,40].

The virtually designed and planned data can be converted into a physical replica by direct or indirect techniques. The direct technique involves the binder jet system to print prototypes models or direct printing of silicone prosthesis, while the indirect technique leads to the fabrication of molds for the packing and vulcanization of silicone. The former technique has the drawback of “pixilation’’ or ‘‘stair-stepping” caused by the thickness of layers, while the deposition of printing material can be partially controlled by orienting the stereolithography (STL) model parallel to the planned prosthesis. The printed model or prosthesis by direct technique is monotonous, which can be masked with manual staining and sealing procedures. The latter technique has the advantage of fabricating the silicone prosthesis from a virtually designed mold with better color matching for a patient-specific skin tone. The fabrication of maxillofacial prostheses and models has been attempted through CAD/CAM techniques with acceptable results, however technical limitations of digital workflow are preventing the direct manufacturing of definitive prostheses for patients. [47,48]. Recently, the development of direct printable silicone has been reported [49,50]. However, reports about its clinical application are lacking.

Further digital advancement is now leading the surgeons toward intra-operative image-guided navigation. Stereotactic navigation during craniofacial bone-anchored implant placement eliminates the need for a physical surgical guide and gives surgeons the ability to simultaneously work within the anatomical defect while being guided through radiographic data in real time [51,52,53]. Bell [54] described registration as “the process of correlating the anatomic references to the digitized dataset”. There are various methods of registering the patient intraoperatively to establish communication with the navigational system. Invasive registration methods require the placement of fixed markers on the patient’s head through small incisions on the scalp or by immobilizing the head and attaching the registration device to a neurosurgical head frame. Noninvasive registration methods include “point registration” through various landmarks on the face, “three-dimensional surface matching” correlating scanned points with the CT, or using a flexible soft tissue–supported adhesive mask that is embedded with light-emitting diodes (LEDs). Currently, two major real-time navigation systems (Stryker and iPlan) are available, which have been used in a few case studies [20,51,55,56]. These navigation systems have never been used to plan craniofacial implants for nasal prostheses; however, their use can be beneficial while placing implants in the floor of the nose due to the proximity of roots of anterior teeth.

A total of 36 implants were placed after digital planning. The surgical templates were designed and printed in 10 cases to assist implants placement intra-operative; however, only two studies assessed the accuracy of the virtual implants position in relation to the final location of implant after placement. According to these studies, the angular deviation ranged from 1.87° to 7.78°, while the apex deviation ranged from 1.17 mm to 3.39 mm for nasal implants [9,19].

Digital planning and designing software have demonstrated the predictable outcomes in numerous case studies by providing viable alternative techniques for rehabilitation of nasal defects (Table 4). It has been evident from the literature that the digital workflow reduces the clinical and laboratory time when compared with conventional procedures while designing and fabricating nasal prosthesis [38] (Table 5). Moreover, the digital software and 3D-printing systems can help to design and fabricate the surgical templates, molds, and prostheses in acceptable time and cost, providing alternative options to conventional techniques [57]. (Table 6) According to Nusair et al. [38], the digital workflow for the fabrication of nasal prosthesis from scanning to delivery of nasal prosthesis took only 5 h, which was significantly less than the conventional technique, which normally takes more than 8 h, to fabricate silicone prostheses (Table 5). Similarly, Ciocca et al. [25] claimed that the design and fabrication of a mold for silicone packing to fabricate nasal prosthesis was completed in 6 h and 22 min, which if fabricated conventionally would take more than 4 h. Additionally, patients can virtually look at the possible outcomes during the planning and designing stages, which can help clinicians and patients to mutually reach the satisfactory outcome [35]. In spite of predictable outcomes and time saving solutions, the equipment cost and technical skills are the limitations, which need to be addressed. Furthermore, the 3D-printed silicone facial prosthesis is another area of future research. According to Unkovskiy [29], currently, the marginal thickness of printed silicone prosthesis can be kept at 0.4 mm, which is thicker than conventionally processed prosthesis with a marginal thickness of 0.1 mm, which makes it difficult to blend the margins of prostheses with adjacent skin. Future digital systems improvement might be able to solve the marginal thickness problem. Additionally, printing silicone prostheses with color matching has not been reported yet due to the limitations of the silicone printing system to exactly match the skin tone of patients.

This review highlighted the digital workflow involved in planning the craniofacial implants locations, designing of molds, substructures, customized implants, resin models, and direct printing of silicone and resin prostheses. It has been evident from the available literature that these computer-assisted software provided predictable outcomes for the rehabilitation of nasal defects; however, this study has the limitation of lack of clinical trials about the assessment of accuracy of these digital software. Meanwhile, this review would open the door for further research to overcome the highlighted problems and limitations.

## 5. Conclusions

Despite the limitations of the assessed literature, digital technology has been increasingly used for various maxillofacial prosthodontics applications and specifically for nasal defects rehabilitation. These applications include defects scanning, virtual design and fabrication of surgical stents for implants placement, fabrication of molds for silicone packing and vulcanization, design of customized implants and retentive attachments and direct printing of silicone nasal prostheses. However, planning with digital software for nasal defects rehabilitation is the most reliable phase of the digital workflow, which saves clinical and laboratory time, reduces patient’s visits, and provides predictable outcome, but equipment costs still pose limitations. The direct printing of silicone nasal prostheses is limited by color formulation and marginal thickness. Further technical development and research is needed to overcome the highlighted limitations.

## Figures and Tables

**Table 1:** Digital planning for craniofacial implants placement and fabrication of nasal prostheses.

Author	Prosthesis	Nº of Cases	Purpose of Software Planning	Pre-Op Data for Digital Planning	Software	Printer/Miller	Printing Materials	Navigation System (Yes/No)	Location and Nº of Implants	Implants System	Post-Op Evaluation
Ciocca et al. 2011 [9]	Nasal prosthesis	1	Surgical template for implants placement	CT, laser scan; NextEngine Desktop ^8^	NobelGuide software ^1^, Amira 3.1.1 software ^2^, Rapidform XOS2 ^3^, Rhino 3.0 ^4^	Stratasys ^21^	Acrylonitrile butadiene styrene plastic material (ABS P400)	No	Glabella; 1 implant. Pre-maxilla; 2 implants	Implants (Branemark System RP TiUnite, Nobel Biocare)	CT scan
Van der Meer et al. 2012 [19]	Nasal prosthesis	3	Surgical template for implants placement	CBCT, Lava COS intraoral scanner ^14^	Mimics software ^5^, 3ds Max ^6^, Geomagic Studio ^7^	DSM Desotech	Biocompatible SLA resin (BioSure, DSM Desotech)	No	Maxilla; 2 implants/defect site. 6 implants in 3 patients	Brånemark, Nobel Biocare	CBCT
Ciocca et al. 2010 [23]	Nasal prosthesis	1	Fabrication of mold for nasal prosthesis	CT, laser scan; NextEngine ^8^	Next Engine software ^8^, Rapidform software ^3^	Stratasys ^21^	Acrylonitrile butadiene styrene (ABS)	No	Maxilla; 2 implants	MKIII TiUnite Nobel Biocare	None
Walivaara et al. 2011 [24]	Nasal prosthesis	1	Surgical template for implants placement	CT	SimPlant Planner 9.2 ^9^	NM	NM	No	Zygomatic bone; 2 implants, Nasofrontal bone; 1 implant	Branemark implants (Brånemark Integration Inc.)	None
Ciocca et al. 2010 [25]	Nasal prosthesis	1	Fabrication substructure for eyeglasses and mold for nasal prosthesis	Laser scan; NextEngine Desktop ^8^, Laser surface scan VIVID 900 ^15^	Rapidform XOS software ^3^, Rhino 4.0 ^4^	Stratasys ^21^	Acrylonitrile butadiene styrene (ABS)	No	No implants	No implants	None
Toso et al. 2015 [26]	Nasal prosthesis	1	Fabrication of nasal implant	CT	3D-modeling software, ZBrush ^10^	NM	Titanium alloy Ti6Al4V	No	Glabella and lateral process of maxilla; customized implant; 1	KLS Martin Group, Tuttlingen, Germany	None
Buzayan et al. 2017 [27]	Nasal prosthesis	1	Surgical template for implants placement	CBCT	Software (Corel PaintShop Pro X4 version 14.0.0.322), Simplant software ^9^	NM	NM	No	Maxilla; 2 implants	Implatium; Bone Level	None
Dawood et al. 2012 [28]	Nasal prosthesis	1	Surgical template for implants placement, Designing and manufacturing of bifunctional implants	NM	Nobel Guide, Nobel Biocare ^1^	NM	Type IV titanium	No	Maxilla; 2 implants	NM	None
Unkovskiy et al. 2018 [29]	Nasal prosthesis	1	Direct printing of silicone prosthesis	Stationery 3D photogrammetry system: pritiface; pritidenta ^16^, light scanner: Artec Spider; Artec 3D	Zbrush Software ^10^	Printer (Drop-on-Demand ACEO; Wacker Chemie AG)	Silicone free of solvents (ACEO Silicone General Purpose; Wacker Chemie AG)	No	Nasal cavity floor; 3 implants	Vistafix 2; Cohlear Ltd.	None
McHutchion et al. 2019 [30]	Nasal prosthesis	1	Surgical template for implants placement, Nose prosthesis prototype and substructure	CBCT	Mimics ^5^, Geomagic Freeform Plus ^7^, Software ZBrush software ^10^	Fortus 400mc; Stratasys ^21^	Thermoplastic material (PC-ISO; Stratasys, Ltd.), thermoplastic material (ABSplus-P430; Stratasys, Ltd.)	No	Right and left zygoma; 2 implants. Right and left lateral maxilla; 2 implants. Glabella; 1 implant	Branemark Systems; Nobel Biocare, Southern Implants	None
Qiu et al. 2011 [31]	Nasal prosthesis	1	Fabrication of mold for nasal prosthesis	CT	Mimics software ^5^, Geomagic Studio 9.0 ^7^	Stereolithography unit (RS4500)	Photopolymer (WaterShed XC, DSM Somos, Elgin, IL, USA)	No	No implants	No implants	None
Reitemeier et al. 2013 [32]	Nasal prosthesis	1	Creation of digital nose database	3D Scan; G scan IVB ^17^	epiTecture software ^13^, Geomagic studio 9.0 ^7^	NM	NM	No	No implants	No implants	None
Grant et al. 2015 [33]	Nasal prosthesis	1	Fabrication of mold for nasal prosthesis	Digital image; 3dMDcranial system ^20^	Materialise: Free-form software ^12^	Binder jetting technique (ProJet 460 plus; 3D Systems)	Cyanoacrylate resin (Apollo 5005 Cyano- acrylate; Cyberbond)	No	No implants	No implants	None
Ciocca et al. 2016 [34]	Nasal prosthesis	1	Designing of substructure	3D laser scanner; 3dMDface System ^19^	Freeform Modeling Plus software and Phantom Desktop Haptic device ^12^	Eosint P100 Formiga ^22^	Polyamide resin and laser-melted cobalt-chrome framework	No	No implants	No implants	None
Palousek et al. 2014 [35]	Nasal prosthesis	1	Designing and rapid prototyping of nose model	3D scan, light scanner; ATOS III ^18^	Rhinoceros software ^4^	ZPrinter 310 Plus ^23^	NM	No	No implants	No implants	None
Dawood et al. 2017 [36]	Nasal prosthesis	1	Fabrication of surgical guide, milling of titanium overdenture bar	CBCT	Nobel- Clinician; NobelBiocare. Procera over-denture bar; Nobel Biocare)	Nobel Biocare	NM	No	No implants	No implants	None
Neto et al. 2014 [37]	Nasal prosthesis	1	Fabrication of mold for nasal prosthesis	CT scans, light scanner; ATOS III ^18^	Mimics 16.0 ^5^	SL-stereolithography; Viper^TM^ SLA System ^24^	NM	No	No implants	No implants	None
Nuseir et al. 2019 [38]	Nasal prosthesis	1	Direct printing of nasal prosthesis	CT scan	Materialise software (CMF Pro Plan ^11^, ZBrush software ^10^	Stratasys ^21^	TangoPlus (Stratasys Ltd.)	No	No implants	No implants	None
Vera et al. 2014 [39]	Nasal prosthesis	1	Fabrication of CAD/CAM copy milled framework for nasal prosthesis	NM	NM	NM	Acrylic resin	No	Anterior maxilla and glabella. Total 3 implants	Vistafix System, Cochlear Corp, Englewood, CO	None
Yoshioka et al. 2016 [40]	Nasal prosthesis	1	Surgical template and model for fabricating substructure	CT, 3D scan Rexcan 3 ^25^	Software (Geomagic Studio ^7^, FreeForm Modeling software ^12^	3D milling machine MDX-40 ^26^	Dental stone New Plastone 2 ^27^	No	No implants	No implants	None
Tso et al. 2015 [41]	Facial prosthesis	1	Fabrication of surgical template	CT scan	NobelGuide ^1^	CAD/CAM machine (Röder RXD5)	NM	No	Right zygoma, left zygoma, right infraorbital rim, left infraorbital rim, nasal and right tuberosity. Total 6 implants	Nobel Biocare	None

Abbreviations: CT: computed tomography; CBCT: cone beam computed tomography; Pre-op: Preoperative; Post-op: Post-operative; NM: Not mentioned; ^1^. NobelGuide, Nobel Biocare, Kloten, Switzerland; ^2^. Amira, Mercury Computer Systems, Chelmsford, MA, USA; ^3^. Rapidform INUS Technology, Seoul, Korea; ^4^. Robert McNeel & Associates, Seattle, WA, USA; ^5^. Materialise NV, Leuven, Belgium; ^6^. Autodesk Inc, San Rafael, CA, USA; ^7^. Geomagic, Morrisville, NC, USA; ^8^. NextEngine, Santa Monica, CA, USA; ^9^. SimPlant, Dentsply implant Hasselt Belgium; ^10^. ZBrush Software; Pixologic Inc., Los Angeles, CA; ^11^. CMF Pro Plan; Materialise, Leuven, Belgium; ^12^. Geomagic Sensable group, Wilmington, MA, USA; ^13^. epiTecture IVS Technology GmbH, Chemnitz, Germany; ^14^. LAVA C.O.S. system; 3M ESPE, Seefeld, Germany; ^15^. Konica Minolta Sensing, Inc, Osaka, Japan; ^16^. Pritidenta GmbH Leinfelden-Echterdingen Germany; ^17^. G-scan; IVB GmbH Jena, Germany; ^18^. ATOS SO, GOM mbH, Braunschweig, Germany; ^19^. 3dMDface System; 3dMD Ltd., London, UK; ^20^. 3dMDcranial System; 3dMD Ltd., Atlanta, GA, USA; ^21^. Stratasys Ltd., Eden Prairie, MN, USA; ^22^. Electro Optical Systems GmbH, Munich, Germany; ^23^. Z Corporation, Burlington, MA, USA; ^24^. 3D Systems Corporation, Rock Hill, SC, USA; ^25^. Rexcan 3; Solutionix Co., Seoul, South Korea; ^26^. MDX-40; Roland DG, Shizuoka-ken, Japan; ^27^. New Plastone 2; GC Corp., Tokyo, Japan.

**Table 2:** Risk of bias for case reports.

Assessment	Author and Year						
	Ciocca et al. 2011 [9]	Ciocca et al. 2010 [23]	Walivaara et al. 2011 [24]	Ciocca et al. 2010 [25]	Toso et al. 2015 [26]	Buzayan et al. 2017 [27]	Dawood et al. 2012 [28]
Were patient’s demographic characteristics clearly described?	Yes	Yes	Yes	Yes	Yes	Yes	No
Was the patient’s history clearly described and presented as a timeline?	Yes	Yes	Yes	Yes	Yes	Yes	No
Was the current clinical condition of the patient on presentation clearly described?	Yes	Yes	Yes	Yes	Yes	Yes	Yes
Were diagnostic tests or assessment methods and the results clearly described?	Unclear	Unclear	Unclear	Unclear	Unclear	Unclear	Unclear
Was the intervention(s) or treatment procedure(s) clearly described?	Yes	Yes	Yes	Yes	Yes	Yes	Yes
Was the post-intervention clinical condition clearly described?	Yes	Yes	Yes	Yes	Yes	No	Yes
Were adverse events (harms) or unanticipated events identified and described?	No	No	No	No	No	No	No
Does the case report provide takeaway lessons?	Yes	Yes	Yes	Yes	Yes	Yes	Yes
Overall appraisal	Included	Included	Included	Included	Included	Included	Included
	**Unkovskiy et al. 2018 [29]**	**McHutchion et al. 2019 [30]**	**Qiu et al. 2011 [31]**	**Reitemeier et al. 2013 [32]**	**Grant et al. 2015 [33]**	**Ciocca et al. 2016 [34]**	**Palousek et al. 2014 [35]**
Were patient’s demographic characteristics clearly described?	Yes	Yes	Yes	No	Yes	No	No
Was the patient’s history clearly described and presented as a timeline?	Yes	Yes	Yes	Yes	Yes	No	No
Was the current clinical condition of the patient on presentation clearly described?	Yes	Yes	Yes	Yes	Yes	Unclear	Yes
Were diagnostic tests or assessment methods and the results clearly described?	Unclear	Unclear	No	Unclear	Unclear	No	No
Was the intervention(s) or treatment procedure(s) clearly described?	Yes	Yes	Yes	Yes	Yes	Yes	Yes
Was the post-intervention clinical condition clearly described?	Yes	Yes	Yes	Yes	Yes	Yes	Yes
Were adverse events (harms) or unanticipated events identified and described?	Yes	No	No	No	No	Yes	No
Does the case report provide takeaway lessons?	Yes	Yes	Yes	Yes	Yes	Yes	Yes
Overall appraisal	Included	Included	Included	Included	Included	Included	Included
	**Dawood et al. 2017 [36]**		**Neto et al. 2014 [37]**	**Nuseir et al. 2019 [38]**	**Vera et al. 2014 [39]**	**Yoshioka et al. 2016 [40]**	**Tso et al. 2015 [41]**
Were patient’s demographic characteristics clearly described?	Yes		Yes	Yes	Yes	Yes	Yes
Was the patient’s history clearly described and presented as a timeline?	Yes		Yes	Yes	Yes	Yes	Yes
Was the current clinical condition of the patient on presentation clearly described?	Yes		Yes	Yes	Yes	Yes	Yes
Were diagnostic tests or assessment methods and the results clearly described?	Unclear		Unclear	Unclear	Yes	Yes	Yes
Was the intervention(s) or treatment procedure(s) clearly described?	Yes		Yes	Yes	Yes	Yes	Yes
Was the post-intervention clinical condition clearly described?	Yes		Yes	Yes	Yes	Yes	Yes
Were adverse events (harms) or unanticipated events identified and described?	No		Yes	No	No	No	No
Does the case report provide takeaway lessons?	Yes		Yes	Yes	Yes	Yes	No
Overall appraisal	Included		Included	Included	Included	Included	Included

**Table 3:** Risk of bias for case series.

Assessment	Author and Year
	Van der Meer et al. 2012 [19]
Were there clear criteria for inclusion in the case series?	Yes
Was the condition measured in a standard, reliable way for all participants included in the case series?	Unclear
Were valid methods used for identification of the condition for all participants included in the case series?	Yes
Did the case series have consecutive inclusion of participants?	Yes
Did the case series have complete inclusion of participants?	Yes
Was there clear reporting of the demographics of the participants in the study?	Unclear
Was there clear reporting of clinical information of the participants?	No
Were the outcomes or follow up results of cases clearly reported?	Yes
Was there clear reporting of the presenting site(s)/clinic(s) demographic information?	Yes
Overall appraisal	Included

**Table 4:** Enlisted are the clinical outcomes, recommendation, and limitations of procedures mentioned in included studies.

Included Articles	Outcome	Recommendations	Limitations
Ciocca et al. 2011 [9]	Post-operative CT scan was done to assess the accuracy of preoperative planning. The implant in glabella had an angular deviation of 7.78° while two implants in premaxilla had an angular deviation of 1.86° and 4.55°. The apex with respect to implants position had deviated by 1.17 mm in glabella, while the implants in premaxilla deviated by 2.81 mm and 3.39 mm, respectively.	The helmet was designed on a rigid and fixed frontal surface of the patient, while the skin is resilient and mobile. Therefore, a bone pin retention system in the future would be better for stabilization of the template.	-
Van der Meer et al. 2012 [19]	Post-operative CBCT was made to analyze the difference between the planned position and actual position of each implant. Assessment revealed that all implants were placed within the limits needed for the fabrication of an optimal prosthesis, both from a surgical and prosthodontic perspective.	-	The slight mismatch between the planned position and actual position of implants may be caused by errors present in the different phases i.e., in the data acquisition phase, the resolution of the CBCT dataset, the accuracy of the system error in the data acquisition of the dentition, integration of the 3D model of the dentition with CBCT dataset errors, and the errors in the polymerization of the SLA material.
Ciocca et al. 2010 [23]	In vivo assessment was done after fabrication of prosthesis. The nasal prosthesis fitted over the defect well. There were no open margins found in the contact region. Furthermore, the connection between the eyeglasses and prosthesis was precise and unambiguous. The fitting was assessed by the Boolean volume difference calculated between the digital models. Furthermore, the mold resulted in a stable, secure position during silicone vulcanization. Considerable less time and cost was involved in manufacturing process.	The use of FDM rapid prototyping systems with thinner layers can improve the final result. Furthermore the silicone adhesive, which is usually used to fix the extrinsic coloring, may help to smooth the surface of the silicone, resulting in a homogenous appearance that eliminates the staircase effect.	The steps are still needed to create software to automate the procedure used to superimpose the model from the digital library onto the digital surface of the defect, to assist in the CAD/CAM bar construction.
Walivaara et al. 2011 [24]	Healing was uneventful. The final nasal prosthesis was retained using magnets attached to the implants.	Author recommends the use of computer-based techniques for planning implants in patients who are exposed to radiation therapy to minimize the need of surgical flaps.	-
Ciocca et al. 2010 [25]	The 3D printer used the FDM technique, which produced a very resistant mold and substructure with ABS material due to stable chemical and thermal properties. Time and cost of fabrication were significantly low	It was suggested that the printing direction should be parallel to the nose to reduce the staircase effect on printed silicone surface. Furthermore, to overcome the staircase effect on silicone surface, silicone adhesive and extrinsic stains can be used to obtain the homogenous surface appearance.	The limitation was the surface roughness produced by the staircase effect caused by the thickness of layers, which were copied in vulcanized silicone.
Toso et al. 2015 [26]	The patient-specific implant was inserted successfully by a paranasal and glabella approach. It fitted precisely three-dimensionally in the preoperative planned position.	Authors recommended the navigation-assisted control of position when typical anatomic reference points are missing.	-
Buzayan et al. 2017 [27]	Prior to the surgical placement of implant, digital planning was done to confirm that the proposed implant positions would not interfere with the future nasal prosthesis margins. That provided the ability to visualize the future prosthetic boundaries and form virtually. As a result, the implants were planned in the anatomical area with the best cosmetic outcome.	-	-
Dawood et al. 2012 [28]	The stability of new bifunctional implants was not enough for immediate loading protocol.	The tissue response of the nasal mucosa to titanium implants or abutments have not been adequately studied or reported. Clinical trials are needed to explore this new approach of simultaneously retaining oral and nasal prosthesis with the bi-functional implant.	There was potential for harmful forces to be transmitted through the retaining superstructure upon the removal of prosthesis.
Unkovskiy et al. 2018 [29]	The directly printed prosthesis was clinically acceptable, which demonstrated the precision and reliability of the digital process. Additionally, the prosthesis was delivered in two visits; thereby, this technique reduced the number of visits of patients	The feasibility of transfer, adaptation, and integration of retaining magnet copings in such prosthesis requires investigation.	The major limitation of this technique was the marginal adaption due to the marginal thickness of 0.4 mm, which could be significantly reduced to below 0.1 mm by the conventional process. The prosthesis was only suitable as an interim postsurgical option for rehabilitation as it was not possible to evaluate the position and marginal adaptation before definitive delivery of the prosthesis.
McHutchion et al. 2019 [30]	Digitally designing the prosthetic components and abutment ensured adequate space for the retentive components without sacrificing the anatomic form. The patient reported satisfaction with the fitting and appearance of the prosthesis upon delivery and at the 4- month follow-up.	The integration of digital technology into the workflow does not necessarily reduce costs as initial investments in computer programs and manufacturing equipment can be costly	Printing directly in a material appropriate for long-term prosthetic use is the critical next step, which can eliminate the need for prototypes and molds.
Qiu et al. 2011 [31]	Due to the geometric complexity, the four-piece mold was rapid prototyped using stereolithography. The prosthesis size, shape, and cosmetic outcome were well accepted by the patient. The prosthesis matched with the nasal defect precise position.	-	The rapid prototyping was carried out by a commercial rapid prototyping center due to the equipment cost, which can be overcome by a cost-effective solution of centralized service.
Reitemeier et al. 2013 [32]	An algorithm was made by using digital nose database to form epiTecture software. The epiTecture software facilitated the virtual positioning of the selected nose from a virtual library by taking into account individual facial asymmetries in the scan. Any type of attachment can be used with a prosthesis fabricated by a digital database. It reduces the laboratory time dramatically, which is normally spent on carving of wax prototype.	Examination of the physical model on the patient is both necessary and practical as the patient’s desires can still be implemented with little effort.	The physical nose model was fabricated with dark colored thermo-polymer at the try in stage, which can be psychologically disadvantageous for patient.
Grant et al. 2015 [33]	The case was done in three sessions of brief physical interaction with the patient and resulted in a well-fitting, esthetic prosthesis. The described process allows the continuous fabrication of prostheses as the child grows, requiring only a 3D digital image that can be used to resize the prosthesis, fabricate a new mold, and process a new prosthesis.	Any device that can capture the midface and provide a file format suitable for 3D design (.stl, .obj, .vrml, .amf, and so on) could be used, including the tissue surface of a computed tomography (CT) scan. This technique can complete the prosthesis in only two visits.	The only limitation is the fracture of mold after the fabrication of two prostheses, which can be overcome by using a different mold material.
Ciocca et al. 2016 [34]	The rapid prototyping technique used in this study enabled perfect transfer of the reciprocal position of the prosthesis with respect to the eyeglasses, from the virtual workflow to the clinical environment. This technique offers improved aesthetics and functional results when no bone is available for implant-supported prostheses.	When a nasal prosthesis has to be stabilized in place through mechanical support (e.g., eyeglasses) rather than implants, long-term follow-up of the connection system is very important.	The limitations of this technique was the final esthetic result, due to the use of eyeglasses and to the difficulty of obtaining a correct profile when a large part of the pre-maxilla was ablated during cancer surgery.
Palousek et al. 2014 [35]	Virtual fitting of a nasal prosthesis before a manufacturing process was possible that enabled the patient and team to evaluate the shape, size and alignment of a nasal prosthesis by 3D visualization. This process led to shortening of manufacturing time and adjustments before insertion of the prosthesis	Authors recommended capturing a digital copy of the nose surface before surgery to get a natural shape. The nose must be replaced by suitable donor geometry.	-
Dawood et al. 2017 [36]	Simultaneous retention of a nasal prosthesis and an intraoral prosthesis was successfully carried out through a custom designed and milled titanium bar with percutaneous nasal extension to retain nasal prosthesis. Planning in software enabled a predictable and straightforward implementation of this novel concept, with the aid of guided surgery.	Although this minimally invasive can provide an option for the prosthetic management of patients for nasal prostheses, tissue engineering options should still be considered.	This approach might be contraindicated if the tissues had been exposed to high-dose radiotherapy post-operatively.
Neto et al. 2014 [37]	Evaluation of the degree of fit was done by distance measurements and a nasal–facial proportion test. Results confirmed the good fit of the nasal prosthesis. This technique saved time and cost along with minimal patient contact.	For the sake of prosthesis’s endurance and hygiene, it is recommended to the patient to have a second prosthesis, which can be fabricated by repeating the last two tasks: prosthesis manufacturing and final fittings.	The limitation of this technique is the inability to reproduce the specificity of some facial features such as delicate skin folds, wrinkles, and textures within prosthesis.
Nuseir et al. 2019 [38]	The final 3D-fabricated nose showed excellent fit over the defect with margins blending seamlessly with adjacent defect tissues. It was due to the printer’s capacity to print 16 μm thick slices. The time taken to manufacture the prosthesis was 5 h with one clinical session.	It was recommended that the printer used in this case has the capability of printing very fine 16-μm-thick slices as compared to the previously presented clinical report where the slice thickness was 400 μm.	The limitation was the color of the prosthesis, which had to be enhanced conventionally.
Vera et al. 2014 [39]	The copy milled CAD/CAM framework was utilized successfully after testing with Sheffield, one screw test for a patient to retain nasal prosthesis. Patient expressed satisfaction with the nasal prosthesis.	For a complex framework, it would be more beneficial to customize the wax pattern manually instead of designing a framework with computer software program.	-
Yoshioka et al. 2016 [40]	The nasal prosthesis was designed using CAD software with the help of presurgical data, which enabled the delivery of an interim nasal prosthesis immediately after rhinectomy.	-	The limitation of this process was inability to provide definitive prosthesis due to unpredictable surgical margins and a continuous healing process.
Tso et al. 2015 [41]	CAD/CAM software was used to fabricate a titanium bar with the Hader bar framework, which retained the obturator and nasal prosthesis. The framework fitted precisely, and patient showed satisfaction at 2 weeks follow up. Patient’s eating and speaking functions were restored after delivery of prostheses.	-	-

**Table 5:** Comparison between conventional and 3D workflows to construct a nasal prosthesis [38].

Procedures	Digital Workflow	
	3D Steps	Conventional Steps
Recording defect	Reconstruction of CT scan	Impression
Time	10	20
Sculpting	Digital design	Manual wax-up (lab) + try in (clinic)
Time (min)	60	120 + 30
Coloring	Digital color production	Silicone mixing and skin tone reproduction
Time (min)	30	60
Nose production	3D printing and post-print processing (including print time)	Flasking and molding, Packing, Curing, Finishing
Time (min)	180	60-30-120-30
External coloring (min)	30	30
Total time (min)	310 (≈5 h)	500 (≈8 h)

**Table 6:** Time and cost estimation for various steps involved during the fabrication of nasal prostheses through digital workflow.

Studies	Purpose	Material	Time	Cost
Ciocca et al. 2010 [23]	Surgical template and drilling steps	ABS	19 h 1 min	64.01 €
Ciocca et al. 2010 [25]	Mold fabrication and substructure	ABS	6 h 22 min	17.10 €
Nuseir et al. 2019 [38]	Scanning, designing, fabrication, and delivery of nasal prosthesis	TangoPlus	5 h	Not mentioned
Neto et.al. 2014 [37]	Prosthesis designing, mold fabrication, prosthesis manufacturing, and delivery of prosthesis	Silicone VTX950	1299 min	651€
Unkovskiy et al. 2018 [29]	Scanning, designing, printing, and manual post-processing	ACEO Silicone	12 h 30 min	Not mentioned

## Data Availability

Generated data is publicly available and cited in accordance with Journal guidelines.
